# Partial Classifier Chains with Feature Selection by Exploiting Label Correlation in Multi-Label Classification

**DOI:** 10.3390/e22101143

**Published:** 2020-10-10

**Authors:** Zhenwu Wang, Tielin Wang, Benting Wan, Mengjie Han

**Affiliations:** 1 Department of Computer Science and Technology, China University of Mining and Technology, Beijing 100083, China; zqt1800405103g@student.cumtb.edu.cn; 2 School of Software and IoT Engineering, Jiangxi University of Finance & Economics, Nanchang 330013, China; wanbenting@jxufe.edu.cn; 3 School of Technology and Business Studies, Dalarna University, 79188 Falun, Sweden

**Keywords:** multi-label classification, classifier chains, label correlation, feature selection

## Abstract

Multi-label classification (MLC) is a supervised learning problem where an object is naturally associated with multiple concepts because it can be described from various dimensions. How to exploit the resulting label correlations is the key issue in MLC problems. The classifier chain (CC) is a well-known MLC approach that can learn complex coupling relationships between labels. CC suffers from two obvious drawbacks: (1) label ordering is decided at random although it usually has a strong effect on predictive performance; (2) all the labels are inserted into the chain, although some of them may carry irrelevant information that discriminates against the others. In this work, we propose a partial classifier chain method with feature selection (PCC-FS) that exploits the label correlation between label and feature spaces and thus solves the two previously mentioned problems simultaneously. In the PCC-FS algorithm, feature selection is performed by learning the covariance between feature set and label set, thus eliminating the irrelevant features that can diminish classification performance. Couplings in the label set are extracted, and the coupled labels of each label are inserted simultaneously into the chain structure to execute the training and prediction activities. The experimental results from five metrics demonstrate that, in comparison to eight state-of-the-art MLC algorithms, the proposed method is a significant improvement on existing multi-label classification.

## 1. Introduction

In machine learning applications, the traditional single label classification (SLC) problem has been explored substantially. However, more recently, the multi-label classification (MLC) problem has attracted increasing research interest because of its wide range of applications, such as text classification [1,2], social network analysis [3], gene function classification [4], and image/video annotation [5]. With SLC, one instance only belongs to one category, whereas with MLC, it can be allocated to multiple categories simultaneously. MLC is a generalization of SLC, which makes it a more difficult and general problem in the machine learning community. Due to multiple labels and the possible links between them, multi-label correlations become very complex [6]. On the one hand, for example, it is more likely for a piece of news tagged with “war” to have another tag “army” than “entertainment”. On the other hand, in a classification of nature scenes with the set of picture labels (“beach”, “building”, “desert”, “sailboat”, “camel”, “city”), it is less likely that a picture of scenery is labelled by both “desert” and “beach”. Thus, exploring these complex couplings is an important challenge in MLC since label correlations can improve classification performance. Based on the order of correlations, the exploitation of label correlations can be divided into roughly three categories [7]:

(1) First-order strategy: This divides the MLC problem into a number of independent binary classification problems. The prominent advantage is their conceptual simplicity and high efficiency even though the related classifiers might not acquire the optimal results because of ignoring the label couplings.
(2) Second-order strategy: This considers pairwise relationships between labels. The resulting classifiers can achieve good levels of generalization performance since label couplings are exploited to some extent. However, they are only able to exploit label-coupling relationships to a limited extent. Many real-world applications go beyond these second-order assumptions.
(3) High-order strategy: This tackles the MLC problem by considering high-order relationships between labels. This strategy is said to have stronger correlation-modeling capabilities than the other two strategies, and its corresponding classifiers to have a higher degree of time and space complexity.

Broadly speaking, there are many relevant works discussing coupling learning of complex interactions [7]. Using a data-driven approach, Wang et al. [8,9] showed how complex coupling relationships could demonstrate learning on categorical and continuous data, respectively, including intra-coupling within objects and inter-coupling between objects. Complex coupling relationships have also been discussed in different applications, such as clustering [10], outlier detection [11] and behavior analysis [12]. Aiming to address the MLC problem, the classifier chain (CC) [13] is a well-known method that adopts a high-order strategy to extract label couplings. Its chaining mechanism allows each individual classifier to incorporate the predictions of the previous one as additional information. However, CC suffers from two obvious drawbacks: (1) the label order is randomly inserted into the chain, which usually has a strong effect on classification performance [14]; (2) all of the labels are inserted into the chain under the assumption that they each have coupling relationships when, in fact, this assumption is too idealistic. Irrelevant labels presenting in the chain actually reduce the predictive results of the CC approach. In this work, we will address the two problems identified here simultaneously. We propose a partial classifier chain method with feature selection (PCC-FS) that exploits the coupling relationships in the MLC problem. The main contributions of this paper include:

(1) A new construction method of chain mechanism that only considers the coupled labels (partial labels) and inserts them into the chain simultaneously, and thus improves the prediction performance;
(2) A novel feature selection function that is integrated into the PCC-FS method by exploiting the coupling relationships between features and labels, thus reducing the number of redundant features and enhancing the classification performance;
(3) Label couplings extracted from the MLC problem based on the theory of coupling learning, including intra-couplings within labels and inter-couplings between features and labels, which makes the exploration of label correlation more comprehensive.

The rest of this paper is organized as follows. The background and reviews of the related work about CC and feature selection in the MLC problem are discussed in Section 2. Based on our reviewed previous research, we outline our PCC-FS approach in Section 3. This consists of three components: feature selection with inter-coupling exploration, intra-coupling exploration in label set, and label set prediction. In Section 4, we discuss the experiment’s environment, the datasets, the evaluation criteria, and the analysis based on the experimental results. Conflicting criteria for algorithm comparison and a series of statistical tests have been carried out for validating the experimental results in Section 5. We conclude the paper in Section 6 by identifying our contribution to this particular research area and our planned future work in this direction.

## 2. Preliminaries

In this section, we begin by introducing the concepts of MLC, CC, and feature selection. This will establish the theoretical foundation of our proposed approach. Then, we will review the CC-based MLC algorithms.

### 2.1. MLC Problem and CC Approach

MLC is a supervised learning problem where an object is naturally associated with multiple concepts. It is important to explore the couplings between labels because they can improve the prediction performance of MLC methods. In order to describe our algorithm, some basic aspects of MLC and CC are outlined first. Suppose (x,y) represents a multi-label sample where x is an instance and y⊆L is its corresponding label set. L is the total label set, which is defined as follows:

(1) $$L=\left\{l_{1},l_{2},\cdots ,l_{Q}\right\},\mathrm{where}\;Q\;\mathrm{is}\;\mathrm{the}\;\mathrm{total}\;\mathrm{number}\;\mathrm{of}\;\mathrm{labels}.$$

We assume that $x=\left(x^{1},x^{2},\cdots ,x^{D}\right)\in X$ is the D-dimensional feature vector corresponding to x, where $X\subseteq R^{D}$ is the feature vector space and $x^{d}\;\left(d=1,\;2,\cdots ,D\right)$ denotes a specific feature. $y=\left(y^{1},y^{2},\cdots ,y^{Q}\right),\in {\left\{0,1\right\}}^{Q}$ is the *Q*-dimensional label vector corresponding to y, and $y^{q}$ is described as:

(2) $$y^{q}=\{\begin{array}{c} 0,\;l_{q}\notin y\\ 1,\;l_{q}\in y \end{array},\;q=1,2,\cdots ,Q.$$

Thus, the multi-label classifier h can be defined as:

(3) $$h:\;X\to {\left\{0,1\right\}}^{Q}.$$

We further assume that there are m+n samples, in which m samples form the training set $X_{train}$ and n samples form the test set $X_{test}$. They are defined as follows:

(4) $$X_{train}=\left\{\left(x_{i},y_{i}\right)\;|\;x_{i}\in X\;and\;y_{i}\in {\left\{0,1\right\}}^{Q},i=1,\;2,\ldots ,m\right\},\;X_{test}=\left\{\left(x_{i},y_{i}\right)|\;x_{i}\in X\;and\;y_{i}\in {\left\{0,1\right\}}^{Q},\;i=m+1,m+2,\ldots ,m+n\right\}.$$

Among the MLC algorithms, CC may be the most famous MLC method concerning label correlations. It involves Q binary classifiers as in the binary relevance (BR) method. The BR method transforms MLC into a binary classification problem for each label and it trains Q binary classifiers $C_{j},\;j=1,\;2,\ldots ,Q$. In the CC algorithm, classifiers are linked along a chain where each classifier handles the BR problem associated with $l_{j}\in L,\;j=1,\;2,\ldots ,Q$. The feature space of each link in the chain is extended with the 0 or 1 label associations of all previous links. The training and prediction phases of CC are described in Algorithm 1 [13]:

**Algorithm 1.** The training phase of the classifier chain (CC) algorithm**Input**: $X_{train}$ **Output**: $C_{j},\;j=1,2,\ldots ,Q$ **Steps** for j∈1, 2, …, Q;do  /*the *j*th binary transformation and training*/;$X_{train}^{\prime }\to \{\}$;for $\left(x_{i},y_{i}\right)\in \;X_{train}$, i=1, 2, …, m;do $X_{train}^{\prime }\leftarrow X_{train}^{\prime }\;U\left\{\left(\left(x_{i},\;l_{1},\ldots ,l_{j-1}\right),l_{j}\right)\right\}$;$C_{j}:X_{train}^{\prime }\to l_{j}\in \left\{0,1\right\}$ /*train $C_{j}$ to predict binary relevance of $l_{j}$ */.

After the training step, a chain $C_{1},\ldots ,C_{Q}$ of binary classifiers is generated. As shown in Algorithm 2, each classifier $C_{j}$ in the chain learns and predicts the binary association of label $l_{j}$, j=1,2,…,Q, augmented by all prior binary relevance predictions in the chain $l_{1},\ldots ,l_{j-1}$.

**Algorithm 2**. The prediction phase of the CC algorithm**Input**: $X_{test}$, $C_{j},\;j=1,2,\ldots ,Q$ **Output**: predicted label sets of each instance in $X_{test}$ **Steps** for $x_{i}\in \;X_{test}$, i=m+1,…,m+n;do;  $y_{i}^{\prime }\leftarrow \{\}$;for j=1 to Q;do $y_{i}^{\prime }\leftarrow y_{i}^{\prime }\;U\left\{\left(l_{j}\leftarrow C_{j}:\left(x_{i},l_{1},\ldots ,l_{j-1}\right)\right)\right\}$;return $\left(x_{i},y_{i}^{\prime }\right)$·i=m+1,…,m+n/*the classified test samples */.

The chaining mechanism of the CC algorithm transmits label information among binary classifiers, which considers label couplings and thus overcomes the label independence problem of the BR method.

### 2.2. Feature Selection in the MLC Problem

Some comprehensive literature reviews and research articles [15,16,17,18] have discussed the problem of feature selection (FS) in the MLC problems. There are different methods used to select relevant features from the MLC datasets. They can be divided into three main types: filters, wrappers, and embedded methods. Filter methods rank all the features with respect to their relevance and cut off the irrelevant features according to some evaluation function. Generally, filter methods adopt an evaluation function that only depends on the properties of the dataset and hence are independent of any particular learning algorithm. Wrappers use an MLC algorithm to search for and evaluate relevant subsets of features. Wrappers usually integrate a search strategy (for example, genetic algorithm or forward selection method) to reduce the high computational burden. For embedded methods, FS is an integral element of the classification process. In other words, the classification process itself performs feature selection as part of the learning process. Therefore, the proposed FS method can be included in the group of filter methods. It adopts covariance to express the coupling relationships between feature set and label set and adaptively cuts off irrelevant features according to the standard deviation of Gaussian-like distribution.

### 2.3. Related Work of CC-Based Approaches

The CC algorithm uses a high-order strategy to tackle the MLC problem; however, its performance is sensitive to the choice of label order. Much of the existing research has focused on solving this problem. Read et al. [19] proposed using the ensemble of classifier chains (ECC) method, where the CC procedure is repeated several times with randomly generated orders and all the classification results are fused to produce the final decision by the vote method. Chen et al. [20] adopted kernel alignment to calculate the consistency between the label and kernel function and then assigned a label order according to the consistency result. Read et al. [21] presented a novel double-Monte Carlo scheme to find a good label sequence. The scheme explicitly searches the space for possible label sequences during the training stage and makes a trade-off between predictive performance and scalability. Genetic algorithm (GA) was used to optimize the label ordering since GA has a global search capability to explore the extremely large space of label permutation [22,23]. Their difference is that the one of the works [23] adopts the method of multiple objective optimization to balance the classifier performance through considering the predictive accuracy and model simplicity. Li et al. [24] applied the community division technology to divide label set and acquire the relationships among labels. All the labels are ranked by their importance.

Some of the existing literature [25,26,27,28,29,30,31,32,33] adopted Graph Representation to express label couplings and rank labels simultaneously. Sucar et al. [25] introduced a method of chaining Bayesian classifiers that integrates the advantages of CC and Bayesian networks (BN) to address the MLC problem. Specifically, they [25] adopted the tree augmented naïve (TAN) Bayesian network to represent the probabilistic dependency relationships among labels and only inserted the parent nodes of each label into the chain according to the specific selection strategy of the tree root node. Zhang et al. [26] used mutual information (MI) to describe the label correlations and constructed a corresponding TAN Bayesian network. The authors then applied a stacking ensemble method to build the final learning model. Fu et al. [27] adopted MI to present label dependencies and then built a related directed acyclic graph (DAG). The Prim algorithm was then used to generate the maximum spanning tree (MST). For each label, this algorithm found its parent labels from MST and added them into the chain. Lee et al. [28] built a DAG of labels where the correlations between parents and child nodes were maximized. Specifically, they [28] quantified the correlations with the conditional entropy (CE) method and found a DAG that maximized the sum of CE between all parent and child nodes. They discovered that highly correlated labels can be sequentially ordered in chains obtained from the DAG. Varando et al. [29] studied the decision boundary of the CC method when Bayesian network-augmented naïve Bayes classifiers were used as base models. It found polynomial expressions for the multi-valued decision functions and proved that the CC algorithm provided a more expressive model than the binary relevance (BR) method. Chen et al. [30] firstly used the Affinity Propagation (AP) [31] clustering approach to partition the training label set into several subsets. For each label subset, it adopted the MI method to capture label correlations and constructed a complete graph. Then the Prim algorithm was applied to learn the tree-structure constraints (in MST style) among different labels. In the end, the ancestor nodes were found from MST and inserted into the chain for each label. Huang et al. [32] firstly used a k-means algorithm to cluster the training dataset into different groups. The label dependencies of each group were then expressed by the co-occurrence of the label pairwise and corresponding labels were then modeled by a DAG. Finally, the parent labels of each label were inserted into the chain. Sun et al. [33] used the CE method to model label couplings and constructed a polytree structure in the label space. For each label, its parent labels were inserted into the chain for further prediction. Targeting the two drawbacks of the CC algorithm mentioned in Section 1, Kumar et al. [34] adopted the beam search algorithm to prune the label tree and found the optimal label sequence from the root to one of the leaf nodes.

In addition to the aforementioned graph-based CC algorithms and considering conditional label dependence, Dembczyński et al. [35] introduced probability theory into the CC approach and outlined their probabilistic classifier chains (PCC) method. Read et al. [36] extended the CC approach to the classifier trellises (CT) method for large datasets, where the labels were placed in an ordered procedure according to the MI measure. Wang et al. [37] proposed the classifier circle (CCE) method, where each label was traversed several times (just once in CC) to adjust the classification result of each label. This method is insensitive to label order and avoids the problems caused by improper label sequences. Jun et al. [38] found that the label with higher entropy should be placed after those with lower entropy when determining label order. Motivated by this idea, they went on to propose four ordering methods based on CE and, after considering each, suggested that the proposed methods did not need to train more classifiers than the CC approach. In addition, Teisseyre [39] and Teisseyre, Zufferey and Słomka [40] proposed two methods that combine the CC approach and elastic-net. The first integrated feature selection into the proposed CCnet model and the second combined the CC method and regularized logistic regression with modified elastic-net penalty in order to handle cost-sensitive features in some specific applications (for example, medical diagnosis).

In summary, in order to address the label ordering problem, almost all of the published CC-based algorithms adopted different ranking methods to determine a specific label order (by including all of the labels or just a part of them). All of these methods are reasonable, but it is hard to judge which label order (or label ordering method) is the best one for a specific application. Furthermore, some of these studies adopted different methods (for example, MI, CE, conditional probability, co-occurrence, and so on) to explore label correlations, but they only focused on label space; the coupling relationships were insufficiently exploited. In addition, the CC-based algorithms used in these published studies added previous labels into feature space to predict the current label, which resulted in an excessively large feature space, especially for large label sets. Thus, feature selection is a necessary stage in the CC-based algorithms. In this work, we propose a novel MLC algorithm based on the CC method and feature selection which avoids the label ranking problem and exploits the coupling relationships both in label and feature spaces. Section 3 provides a detailed description of the proposed method.

## 3. The Principle of the PCC-FS Algorithm

Inspired by the CCE method [37], the research presented here organizes labels as a circular structure that can overcome the label ordering problem. However, there are two obvious differences between the PCC-FS algorithm and the CCE algorithm. First, the CCE algorithm included all of the labels in the training and prediction tasks while the PCC-FS algorithm only uses coupled labels to perform these tasks. Second, CCE does not take advantage of label correlations while the PCC-FS algorithm not only exploits the intra-couplings within labels but also explores the inter-couplings between features and labels.

### 3.1. Overall Description of the PCC-FS Algorithm

**Figure 1:**
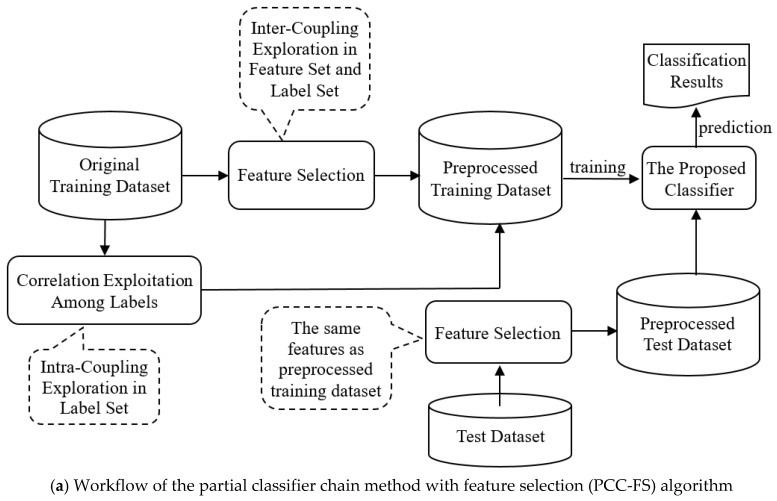

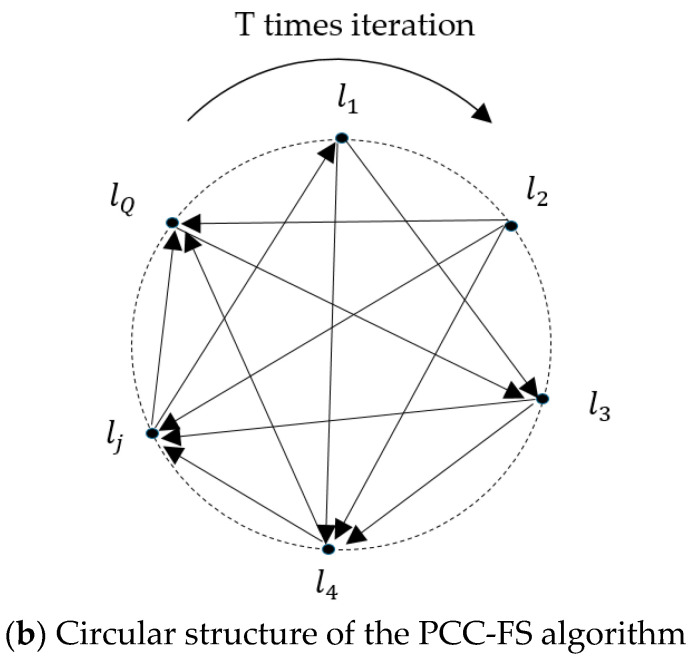
The framework of the PCC-FS algorithm.

The PCC-FS algorithm aims to solve the MLC problem by exploring coupling relationships in feature and label spaces. The workflow is described in Figure 1a and includes the following three steps:

(1) In the feature selection stage, we explore the inter-couplings between each feature and label set. The features with low levels of inter-couplings would be cut off on the assumption that all the inter-couplings follow Gaussian-like distribution;
(2) Intra-couplings among labels are extracted to provide the measurement used to select the relevant labels (partial labels) of each label. Irrelevant labels are not then able to hinder the classification performance;
(3) After feature selection, as described in Figure 1b, the coupled labels of each label are inserted simultaneously into the chain, and label prediction is executed in an iterative process, thus avoiding the label ordering problem.

In order to elaborate on the PCC-FS algorithm in greater detail, we will discuss the above three steps in the following sections. Feature selection, as the data preprocessed step, is discussed in Section 3.2. Intra-coupling exploration is introduced in Section 3.3 and Section 3.4 gives a detailed description of the training and prediction steps in the PCC-FS algorithm.

### 3.2. Feature Selection with Inter-Coupling Exploration

In order to eliminate the differences of the various features, the values of each feature are normalized, which is denoted as $x^{d:norm}$ for d=1, 2, …, D. The PCC-FS algorithm adopts the absolute value of covariance, denoted as $Cov\left(x^{d:norm},l_{j}\right)$, to represent the coupling relationships between normalized feature $x^{d:norm}$ and label $l_{j}$. The reason for adopting absolute covariance is that both positive and negative covariance can indicate the correlation between features and labels. In order to calculate $Cov\left(x^{d:norm},l_{j}\right)$, $l_{j}$ is encoded by binary value (1 for containing label $l_{j}$ and 0 otherwise) (We give a concrete example to illustrate the numerical coding. Suppose that we classify bird species by their acoustic features. One of the methods is to convert the audio signal to a spectrogram, which is further represented by an image. Four sample images are digitalized and vectorized to generate the feature matrix $x^{d:norm}$. The first label of all images—e.g., $l_{1}=\;{\left[\begin{array}{cc} \begin{array}{cc} 0.00 & 0.00 \end{array} & \begin{array}{cc} 1.00 & 1.00 \end{array} \end{array}\right]}^{T}$—has the same dimension to the first feature—e.g., $\begin{array}{c} x^{1:norm} \end{array}=\;{\left[\begin{array}{cc} \begin{array}{cc} 0.00 & 0.33 \end{array} & \begin{array}{cc} 0.67 & 1.00 \end{array} \end{array}\right]}^{T}$. Thus, the unbiased sample covariance will be $Cov\left(\begin{array}{c} x^{1:norm} \end{array},l_{1}\right)=\left|E\left(\begin{array}{c} x^{1:norm} \end{array}*l_{1}\right)-E\left(\begin{array}{c} x^{1:norm} \end{array}\right)E\left(l_{1}\right)\right|=0.22$.). It should be noted that there are many other encoding methods to be developed and our algorithm works only for numerical encoding. The inter-coupling between $x^{d:norm}$ and label set is defined in Equation (5):

(5) $$IeC\left(x^{d:norm},L\right)=\frac{\sum_{j=1}^{Q}Cov\left(x^{d:norm},\;l_{j}\right)}{Q}$$

For every feature, we suppose that all the covariance values between feature set and label set follow the Gaussian-like distribution with a concave density function. Since too small inter-coupling has to be discarded, what we need to figure out is the quantile where we truncate the sample irrespective of the distribution. Thus, we do not restrict them to be exactly in Gaussian distribution. Under this relaxation, we can apply the same criteria described in Equation (6) for the truncation, where μ is estimated by sample mean and σ by sample standard deviation:

(6) $$\mu -IeC\left(x^{d:norm},L\right)\;>\;\sigma .$$

We further suppose that there are m training instances and n test instances. The feature-selected training dataset ${Ƶ}_{train}$ is defined as follows in Equation (7):

(7) $${Ƶ}_{train}=\left(X_{train},Y_{train}\right),\;X_{train}={\left[\begin{array}{ccc} x_{1}^{1} & \ldots & x_{1}^{D^{\prime }}\\ \ldots & x_{i}^{d} & \ldots \\ x_{m}^{1} & \ldots & x_{m}^{D^{\prime }} \end{array}\right]}_{m\times D^{\prime }},\;Y_{train}={\left[\begin{array}{ccc} y_{1}^{1} & \ldots & y_{1}^{Q}\\ \ldots & y_{i}^{j} & \ldots \\ y_{m}^{1} & \ldots & y_{m}^{Q} \end{array}\right]}_{m\times Q}.$$

$X_{train}$ represents the matrix that contains all the feature values of m training instances, and $Y_{train}$ is the matrix of labels for all the training instances. $D^{\prime }$ is the number of dimensions after feature selection. Similarly, the test dataset $X_{test}$ is described in Equation (8):

(8) $$X_{test}={\left[\begin{array}{ccc} x_{1}^{1} & \ldots & x_{1}^{D^{\prime }}\\ \ldots & x_{i}^{d} & \ldots \\ x_{n}^{1} & \ldots & x_{n}^{D^{\prime }} \end{array}\right]}_{n\times D^{\prime }}.$$

### 3.3. Intra-Coupling Exploration in Label Set

In the CC algorithm, it is unreasonable that all of the previous labels participate in the learning activity of the current label because it is a highly idealistic assumption that all of the previous labels couple with the current label. In this study, we use the absolute covariance to measure the intra-couplings among labels, named as $IaC\left(l_{j},l_{k}\right),\;j,k=1,\;2,\ldots ,Q$. The covariance matrix of labels is described in Equation (9):

(9) $$\phi ={\left[\begin{array}{ccc} IaC\left(l_{1},l_{1}\right) & \ldots & IaC\left(l_{1},l_{Q}\right)\\ \ldots & IaC\left(l_{j},l_{k}\right) & \ldots .\\ IaC\left(l_{Q},l_{1}\right) & \ldots & IaC\left(l_{Q},l_{Q}\right) \end{array}\right]}_{Q\times Q}.$$

The threshold method is used to judge the intra-coupling relationships, where the threshold $IaC_{thre}$ is determined by experimental analysis instead of subjective empirical constants. Specifically, the alternative threshold values are sampled K (for example, K=100) groups with equal space in $\left[Max_{IaC},Min_{IaC}\right]$, where $Max_{IaC}$ and $Min_{IaC}$ are the maximum and minimum values in ϕ, excluding the values on the main diagonal. The threshold $IaC_{thre}$ is determined by calculating the comprehensive average ranking across five criteria. According to $IaC_{thre}$, ϕ is transformed into a matrix with only 0 and 1 values, where one indicates that the corresponding value is no less than the threshold and zero denotes the other cases. For example, if the first line of transformed ϕ is ${\left[\begin{array}{ccc} 1 & 1 & 0 \end{array}\cdots \begin{array}{ccc} 0 & 1 & 1 \end{array}\right]}_{1\times Q}$, it indicates that $l_{1}$ only has coupling relationships with $l_{2}$, $l_{Q-1,}$ and $l_{Q}$. For each label $l_{j}$, j=1, 2,⋯, Q, we defined its coupled label set in Equation (10):

(10) $${Ɣ}_{j}\;=\left\{l_{k}|\begin{array}{c} \exists IaC\left(l_{j},l_{k}\right)\in \phi ,\;\\ s.t.\;IaC\left(l_{j},l_{k}\right)\geq IaC_{thre},\\ \;k=1,\;2,\cdots ,\;Q\; \end{array}\right\}.$$

### 3.4. Label Prediction of the PCC-FS Algorithm

In the PCC-FS algorithm, the label set L is organized as a circular structure with random order as described in Figure 1b. For each label $l_{j},\;j=1,\;2,\;\ldots ,Q$, the proposed algorithm constructs binary classifiers by the order of $\left\{l_{1},l_{2},\cdots ,l_{Q}\right\},$ and this process iterates T times. In each iteration, ${Ɣ}_{j}$ is regarded as an additional feature set for the binary classifier related to label $l_{j}$. We suppose that the binary classifier is defined as follows:

(11) $${ƕ}_{r,j}\leftarrow Ɓ\left(\chi_{train}^{r,j}\right),\;r=1,\;2,\ldots ,T;\;j=1,\;2,\ldots ,Q.$$

The PCC-FS algorithm generates T∗Q binary classifiers, ${ƕ}_{r,j}$, and Ɓ represents the binary learning method. In this work, logistic regression is used as the base binary classifier. In addition, other binary learning methods can also be applied to our algorithm. Ƈ contains the latest predicted values of all of the labels on *m* training instances, as described in Equation (12):

(12) $$Ƈ={\left[\begin{array}{ccc} l_{11} & \ldots & l_{1Q}\\ \ldots & l_{jk} & \ldots .\\ l_{m1} & \ldots & l_{mQ} \end{array}\right]}_{m\times Q}.$$

For the first iteration of the training process, Ƈ is initialized by zero matrix ${\left[0\right]}_{m\times Q}$. Ƈ(j) represents the vector that contains the latest predicted values of $l_{j}$ on all the training instances. Ƈ(j) is derived from Ƈ and iteratively updated by ${ƕ}_{r,j}$. Similarly, $Ƈ\left({Ɣ}_{j}\right)$ is the matrix that contains the latest predicted values of ${Ɣ}_{j}$ on all the training instances. $\chi_{train}^{r,j}$ is the binary dataset related to $l_{j}$ in the *r*th iteration, which is defined as follows:

(13) $$\chi_{train}^{r,j}=\left[x^{r,j},Y_{train}\left(j\right)\right],\;$$

and

(14) $$x^{r,j}=\left[X_{train},lpre^{r,j}\right]=\left[X_{train},Ƈ\left({Ɣ}_{j}\right)\right],$$

where r=1, 2,…, T,j=1, 2, …,Q. $Y_{train}\left(j\right)$ is the label vector related to label $l_{j}$ and $x^{r,j}$ is the extended feature matrix. $lpre^{r,j}$ represents the latest predicted results of ${Ɣ}_{j}$, which can be acquired by $Ƈ\left({Ɣ}_{j}\right)$. The training steps of the PCC-FS algorithm is, therefore, described in Algorithm 3.

**Algorithm 3**. The training phase of the PCC-FS algorithm**Input**: original training data, iterative times T **Output**: T∗Q binary classifiers ${ƕ}_{r,j},$
r=1, 2,…,T;j=1, 2,…,Q
**Steps**
generate $X_{train}$ through feature selection process;learn label couplings and generate ${Ɣ}_{j}$ for each label by Equation (14), j=1, 2,…,Q;initialize $\chi_{train}^{1,j}$ with zero matrix ${\left[0\right]}_{m\times Q}$;for r∈1, 2,…, T;for j∈1, 2, …, Q;$lpre^{r,j}=Ƈ\left({Ɣ}_{j}\right)$;$x^{r,j}=\left[X_{train},lpre^{r,j}\right]$;$\chi_{train}^{r,j}=\left[x^{r,j},Y_{train}\left(j\right)\right]$;${ƕ}_{r,j}\leftarrow B(\chi_{train}^{r,j}$);$Ƈ\left(j\right)={ƕ}_{r,j}\left(x^{r,j}\right)$;end for;end for.


As described in Algorithm 3, the PCC-FS algorithm learns ${ƕ}_{r,j}$ and updates $\chi_{train}^{r,j}$ iteratively. The latest prediction results of each label are then integrated into the classifiers ${ƕ}_{r,j}$ to perform the current prediction activities. After iterating T times, the PCC-FS algorithm should acquire T∗Q binary classifiers ${ƕ}_{r,j}$ for r=1, 2,…,T; j=1, 2,…,Q. The prediction steps of the PCC-FS algorithm are described in Algorithm 4 where the trained T∗Q binary classifiers ${ƕ}_{r,j}$ predict the label set for each test sample in an iterative process by using $X_{test}$ and ${Ɣ}_{j}$.

**Algorithm 4**. The prediction phase of the PCC-FS algorithm**Input**: original test data, T∗Q binary classifiers ${ƕ}_{r,j},$
r=1, 2,…,T; j=1, 2,…,Q; **Output**: predicted label sets for $X_{test}$; **Steps**
generate $X_{test}$ through feature selection process;initialize $\chi_{test}^{1,j}$ with zero matrix ${\left[0\right]}_{n\times Q}$;for r∈1, 2,…, T;for j∈1, 2, …, Q;$lpre^{r,j}=Ƈ\left({Ɣ}_{j}\right)$;$\chi_{test}^{r,j}=\left[X_{test},lpre^{r,j}\right]$;$Ƈ\left(j\right)={ƕ}_{r,j}\left(\chi_{test}^{r,j}\right)$;end for;end for;return the predicted results Ƈ.


## 4. Experimental Results and Analysis

Using the introduction of an experimental environment and datasets, this section provides an experimental analysis and comparison of the proposed PCC-FS method and eight other state-of-the-art MLC algorithms.

### 4.1. Experiment Environment and Datasets

We included seven datasets in the experiments conducted for this article, all of which are extensively used to evaluate MLC algorithms. The themes of the data include emotions, CAL500, yeast, flags, scene, birds, and enron (for detailed information about these public datasets please see: http://mulan.sourceforge.net/datasets-mlc.html). They cover text, image, music, audio, and biology fields, and the number of labels varies from 6 to 174, as described in Table 1.

### 4.2. Evaluation Criteria

In this work, five popular MLC criteria were adopted to validate our method including Hamming Loss (HL), Ranking Loss (RL), One Error (OE), Coverage (Cove), and Average Precision (AP). We used f to denote the function of predicted probability. The predicted probabilities that test instance belonging to each label were sorted in descending order, and $rank_{f}\left(x_{i}^{fs},l\right)$ presents the corresponding rank of label l. The symbol |·| in the following criteria indicates the number of the element number in a set. Hamming Loss computes the average number of times that labels are misclassified. Δ is the symmetric difference between two sets:

(15) $$HL\left(h\right)=\frac{1}{n}\overset{m+n}{\underset{i=m+1}{\sum }}\frac{1}{Q}\left|h\left(x_{i}^{fs}\right)\Delta y_{i}\right|.$$

Ranking Loss computes the average number of times when irrelevant labels are ranked before the relevant labels. $\bar{y_{i}}$ is the complement of $y_{i}$ in L:

(16) $$RL\left(h\right)=\frac{1}{n}\overset{m+n}{\underset{i=m+1}{\sum }}\frac{1}{\left|y_{i}\right|\left|\bar{y_{i}}\right|}\left|\left\{\left(l,l^{\prime }\right)\in y_{i}\times \bar{y_{i}}|f\left(x_{i}^{fs},l\right)\leq f\left(x_{i}^{fs},l^{\prime }\right)\right\}\right|.$$

One Error calculates the average number of times that the top-ranked label is irrelevant to the test instance:

(17) $$OE\left(h\right)=\frac{1}{n}\overset{m+n}{\underset{i=m+1}{\sum }}\left|\left\{l\notin y_{i}|l=\arg\underset{l\in L}{\max}rank_{f}\left(x_{i}^{fs},l\right)\right\}\right|.$$

Coverage calculates the average number of steps that are in the ranked list to find all the relevant labels of the test instance:

(18) $$Cove\left(h\right)\;=\;\frac{1}{n}\overset{m+n}{\underset{i=m+1}{\sum }}\underset{l\in y_{i}}{\max}rank_{f}\left(x_{i}^{fs},l\right)-1.$$

Average Precision evaluates the degree for the labels that are prior to the relevant labels and that are still relevant labels:

(19) $$AP\left(h\right)\;=\;\frac{1}{n}\overset{m+n}{\underset{i=m+1}{\sum }}\frac{1}{\left|y_{i}\right|}\underset{l\in y_{i}}{\sum }\frac{\left|\left\{l^{\prime }\in y_{i}|f\left(x_{i}^{fs},l\right)\leq f\left(x_{i}^{fs},l^{\prime }\right)\right\}\right|}{rank_{f}\left(x_{i}^{fs},l\right)}.$$

### 4.3. Experimental Results Analysis and Comparison

Eight state-of-the-art MLC algorithms were chosen for a comparison study in order to act as a contrast to the proposed PCC-FS algorithm. These are HOMER [41], LP [42], RAkEL [43], Rank-SVM [44], BP_MLL [45], CC [13], CCE [37], and LLSF-DL [46]. HOMER, LP, RAkEL, Rank-SVM, and BP_MLL are classic benchmark algorithms. CC and CCE are the CC-based algorithms, and LLSF_DL is a recently developed algorithm whose learning of label-specific data representation for each class label and class-dependent labels has performed outstandingly. For comparative objectivity, 10-fold cross validation was adopted, and the average values of 10 experimental repetitions were regarded as the final values for every evaluation criterion. The base classifier of CC, CCE, and PCC-FS is linear logistic regression, which was implemented by the Liblinear Toolkit. The full names of the compared algorithms in Table 2, Table 3, Table 4, Table 5, Table 6 and Table 7 are as follows: HOMER: hierarchy of multi-label classifiers; LP: label powerset; RAkEL: RAndom k-labELsets; Rank-SVM: rank support vector machine; BP_MLL: back-propagation for multi-label learning; CC: classifier chains; CCE: classifier circle; PCC-FS: partial classifier chains with feature selection; LLSF-DL: Learning Label-Specific Features and Class-Dependent Labels.

**Figure 2:**
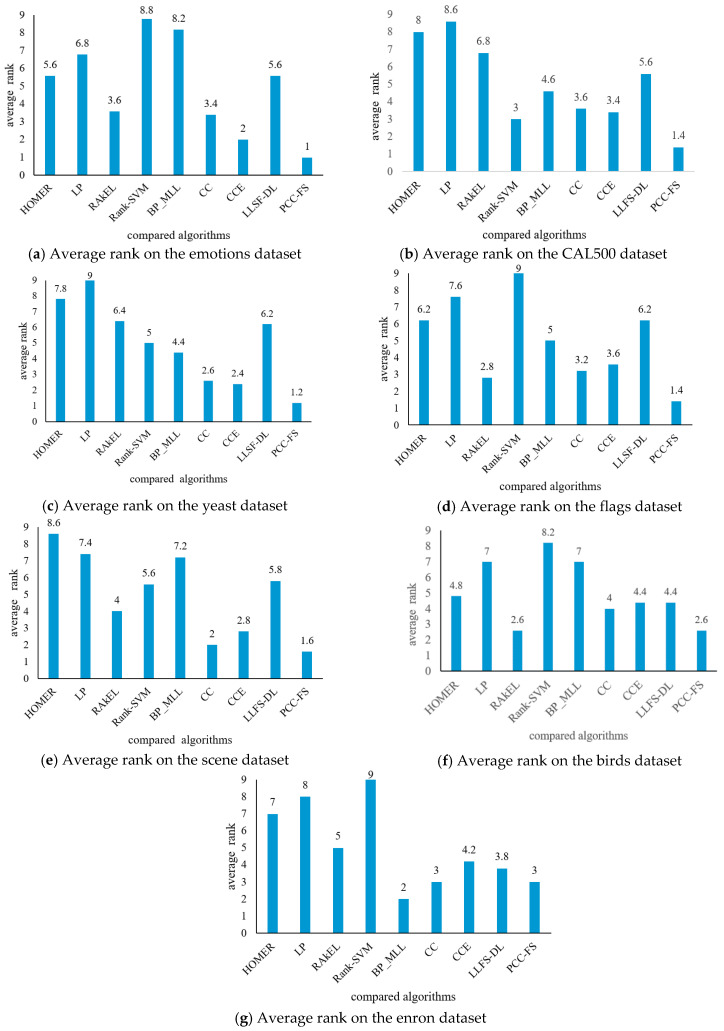
Average rank of nine compared algorithms on different datasets.

Generally speaking, as observed from Table 2, Table 3, Table 4, Table 5, Table 6, Table 7 and Table 8, the LP algorithm demonstrated poor performance across all seven datasets. Five algorithms (Rank-SVM, BP_MLL, HOMER, RAkEL, and LLSF-DL) showed poor performance on some datasets. More specifically, the Rank-SVM algorithm achieved the worst comprehensive performance on the datasets of emotions, flags, birds, and enron. The BP_MLL algorithm did not perform well on the datasets of emotions and scene, while it achieved good results on dataset enron. HOMER’s results were not good on the datasets of yeast, scene, and enron. The RAkEL algorithm did not achieve good performance on dataset yeast, but its performance on dataset flags showed obvious advantages over other algorithms except PCC-FS. The results of LLSF-DL algorithm were not good on the datasets of flags or yeast, besides showing no obvious advantages among the nine algorithms. For dataset scene, the CC algorithm achieved the best results among the nine algorithms on Ranking Loss, Coverage, and Average Precision. The RAkEL algorithm attained the best values for the Hamming Loss criterion but only on the datasets of birds and flags. RAkEL also obtained the best value for the Ranking Loss criterion on dataset birds. For five of the tested evaluation criteria, the proposed PCC-FS algorithm outperformed all of the eight other algorithms with the best comprehensive performance on the datasets of emotions, CAL500, yeast, flags, scene, and birds, and it achieved above-average performance on dataset enron. In order to evaluate the performance of all nine algorithms across these five criteria, their average ranks are presented in Figure 2.

The Rank-SVM algorithm achieved the worst average rank on the datasets of emotions, flags, birds, and enron; the LP algorithm obtained the worst average rank on CAL500 and yeast; and the HOMER algorithm the worst average rank on scene. For our proposed PCC-FS algorithm, its average rank on seven datasets (emotions, yeast, CAL500, flags, scene, birds, and enron) was 1, 1.2, 1.4, 1.4, 1.6, 2.6, and 3, respectively. The comparison results demonstrate that the proposed PCC-FS algorithm achieved stable results and significant classification effects on the seven commonly-used datasets in contrast to the eight other most-cited algorithms.

## 5. Conflicting Criteria for Algorithm Comparison and Statistical Test

### 5.1. Conflicting Criteria

**Figure 3:**
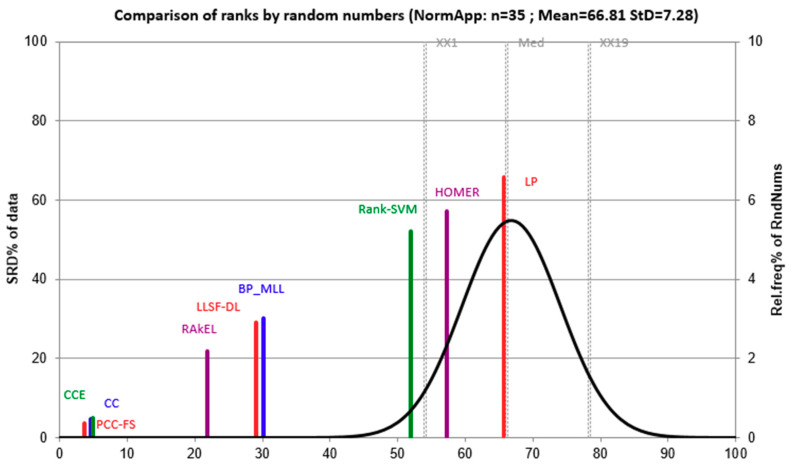
Evaluation of algorithms using sum of ranking differences. Scaled sum of ranking differences (SRD) values are plotted on x-axis and left y-axis, and right y-axis shows the relative frequencies (black curve). Parameters of the Gaussian fit are m = 66.81 s = 7.28. Probability levels 5% (XX1), Median (Med), and 95% (XX19) are also given.

Conflicting criteria may exist when comparing algorithms under multiple evaluation methods, because the five methods used in this work did not give consistent ranking results. To conduct a fair comparison between algorithms, we presented the outcome of the sum of ranking differences (SRDs) that is a multi-criteria decision-making tool before making statistical test [47,48]. The absolute values of differences between a reference vector and actual ranking were summed up for each algorithm. Since we have five evaluation methods and seven data sets, the reference was a vector of 35 elements with each element being the best score across each algorithm. The theoretical distribution for SRD was approximately normal after scaling it onto an interval of [0,100]. Thus, the normal quantile for each algorithm represented an empirical SRD compared with the reference vector. A detailed implementation is also available in a recent work [49]. The comparison was shown in Figure 3.

From Figure 3, we can see that PCC-FS was located to the left of the curve indicating that PCC-FS is the ideal algorithm and the closest one to the reference. Meanwhile, CC and CCE were in the vicinity of PCC-FS, which means that PCC-FS, CC, and CCE are comparable to each other. Moreover, except for HOMER and LP, the remaining seven algorithms were significantly (p=0.05) different from a random ranking by chance. In this sense, we obtained an overview of the group of ideal algorithms and their significance level. Statistical tests and confidence intervals will further quantify the differences.

### 5.2. F-Test for All Algorithms

In this work, we also conducted a Friedman test [50] to analyze performance among the compared algorithms. Table 9 provided the Friedman statistics $F_{F}$ and the corresponding critical value in terms of each evaluation criterion. As shown in Table 9, the null hypothesis (that all of the compared algorithms will perform equivalently) was clearly rejected for each evaluation criterion at a significance level of α=0.05. Consequently, we then proceeded to conduct a post-hoc test [50] in order to analyze the relative performance among the compared algorithms.

**Figure 4:**
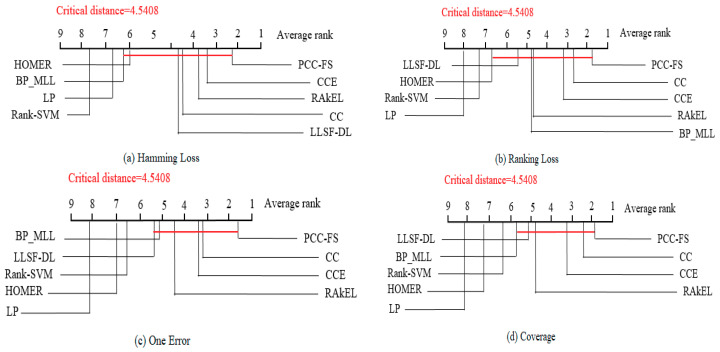

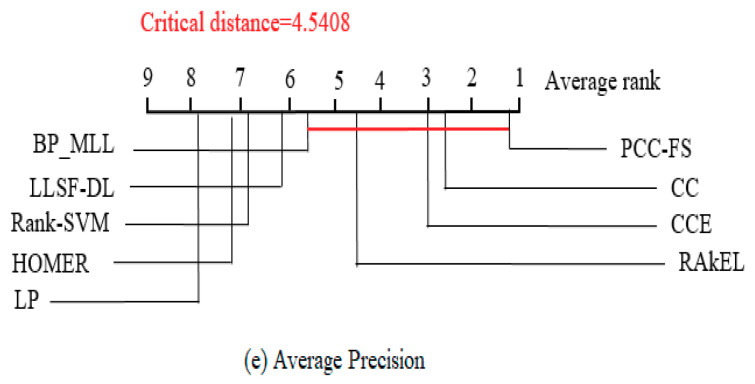
Comparison of PCC-FS (control algorithm) against other compared algorithms using the Nemenyi test.

The Nemenyi test [50] was used to test whether each of the algorithms performed competitively against the other compared algorithms, where PCC-FS was included. Within the test, the performance between two classifiers was considered to be significantly different if the corresponding average ranks differed by at least the critical difference $\mathrm{CD}=q_{\alpha }\sqrt{\frac{k\left(k+1\right)}{6N}}$. For the test, $q_{\alpha }$ is equal to 3.102 at the significance level α=0.05, and thus CD takes the value of 4.5409 (k = 9, N = 7). Figure 4 shows the CD diagrams for each of the five evaluation criteria, with any compared algorithm whose average rank was within one CD to that of PCC-FS connected to it with a red line. Algorithms that were unconnected to PCC-FS were otherwise considered to have a significantly different performance between them. In Hamming Loss, for example, the average rank for PCC-FS was 2.14, and the critical value would be 6.68 by adding CD. Since LP and Rank-SVM got 6.71 and 7.57 for their respect average rankings, they were classified as worse algorithms. However, we could not distinguish the performance of the remaining algorithms from PCC-FS. Since the F-test is based on all algorithms, we will further consider PCC_FS as a control algorithm and make a pairwise comparison in Section 5.3.

### 5.3. PCC-FS as Control Algorithm

In order to increase the power of the test, we also considered PCC-FS as a control algorithm and compared it against all other algorithms. For this, we used Bonferroni correction for controlling the family-wise error or the probability of making at least one Type 1 error in multiple hypothesis tests. The comparison was made by examining the critical difference, $CD_{Bon}$, while considering the Bonferroni correction that is conservative in that the critical value for $q_{\alpha }$ becomes 2.724 when α=0.05. The results for that the average ranking difference, $\Delta \xi ={\bar{\xi }}_{other}-{\bar{\xi }}_{PCC-FS}$, is larger $CD_{Bon}$ are marked by “√” in Table 10. It has already been seen in Section 5.2 that Δξ>0 for all cases. Empty cells indicate that Δξ was within $CD_{Bon}$. From Table 10, we can see that PCC-FS outperforms five algorithms (HOMER, LP, Rank-SVM, BP_MLL, and LLSF-DL) under at least one evaluation criterion.

### 5.4. Confidence Intervals

Confidence interval [51] can further imply how much better it performs when PCC-FS is compared with other algorithms. To quantify the difference, we constructed the intervals for all eight comparisons. The normality assumption [50] was made on the ranking differences:

$$\frac{\Delta \xi }{\sqrt{k\left(k+1\right)/6N}}\sim N\left(0,1\right).$$

**Figure 5:**
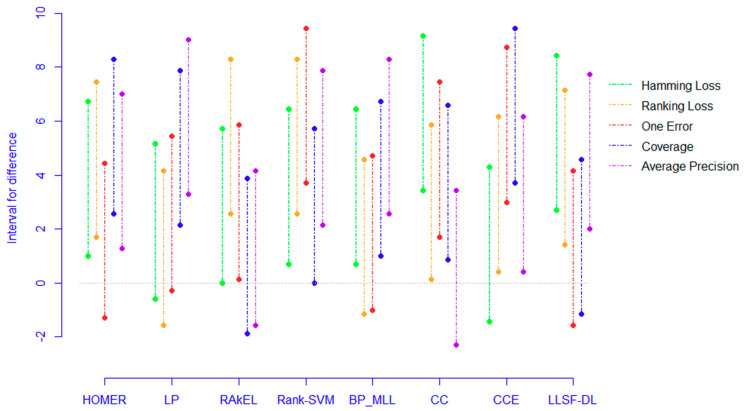
Confidence interval for ranking difference.

Under the 95% confidence level, we show the intervals for each algorithm in Figure 5. Furthermore, five criteria were grouped. Among the five worse algorithms, all intervals for Rank-SVM seemed to be greater than 0, which indicated a significant difference compared to PCC-FS. The extreme upper bound was close to 10 for One Error. For the remaining four, the majority of lower bounds was greater than or close to 0, while the overall upper bounds were slightly less than that of Rank-SVM. For the other three seemingly indifferent algorithms, four out of five intervals for CC and CCE presented positive values and three out of five for RAkEL. Even though some of the criteria indicated a negative lower bound for CC and CCE, the average values for lower bounds were positive. However, RAkEL seemed to be a comparable algorithm to PCC-FS.

### 5.5. Summaries

Based on these experimental results, the following observations can be made:

(1) The proposed PCC-FS algorithm achieves the top average rank among nine algorithms across all five criteria, and the CC-based high-order algorithms (CC, CCE, PCC-FS) in general achieve better performance than the other algorithms. This is because these types of algorithms exploit the label couplings thoroughly.
(2) Four out of five ranking differences for CC and CCE have shown positive intervals meaning that the probability of obtaining a higher rank for PCC-FS compared with CC or CCE is 80% for a given dataset even though they are comparable.
(3) PCC-FS outperforms LP, HOMER, Rank-SVM, BP_MLL, and LLSF-DL because the ranking differences for them are significantly larger than the critical value across the five tested criteria. Corresponding confidence interval gives an overview of the quantified amount in ranking difference.
(4) LP, HOMER, and Rank-SVM perform the worst on all of the five criteria because LP and HOMER transform MLC into one or more single label subproblems. Rank-SVM divides MLC into a series of pairwise classification problems and cannot be seen to describe label couplings very well.
(5) RAkEL performs neutrally among the test algorithms.

## 6. Conclusions

The MLC problem is an important research issue in the field of data mining, which has a wide range of applications in the real world. Exploring label couplings can improve the classification performance of the MLC problem. The CC algorithm is a well-known way to do this. It adopts a high-order strategy in order to explore label correlations, but it does have two obvious drawbacks. Aiming to address both problems at the same time, we proposed the PCC-FS algorithm, which extracts intra-couplings within label sets and inter-couplings between features and labels. In doing so, our new algorithm makes three major contributions to the MLC problem. First, it uses a new chain mechanism which only considers the coupled labels of each label and organizes them to train and predict data and thus improving prediction performance. Second, by integrating a novel feature selection method into the algorithm to exploit the coupling relationships between features and labels, PCC-FS is able to reduce the number of redundant features and improve classification performance. Third, extracting label couplings in the MLC problem based on the theory of coupling learning, including intra-couplings within labels and inter-couplings between features and labels, makes the exploration of label couplings more sufficient and comprehensive. Compared with other testing algorithms, PCC-FS has the best average ranking and CC-based algorithms are comparable. The analytical results given by multi-criteria decision-making and statistical test are consistent. Using confidence intervals for ranking differences further implies how much better PCC-FS has performed.

In the future, some effort will be required to improve and extend the proposed PCC-FS algorithm. First, in our tests, we only used the logistic regression method as the binary classifier. Any further work on the algorithm should investigate the performance of different basic binary classifiers more thoroughly. Second, more adaptive methods of threshold selection should be studied in order to enhance the accuracy and automation of the PCC-FS algorithm. Third, more normalization methods, for example, rank transformation, will be applied to normalize the feature values.

## Figures and Tables

**Table 1:** Description of multi-label datasets.

Dataset	Instance Number	Label Number	Continuous Feature Number	Discrete Feature Number	Density	Field
emotions	593	6	72	0	0.311	Music
CAL500	502	174	68	0	0.150	Music
yeast	2417	14	103	0	0.303	biology
flags	194	7	10	9	0.485	Image
scene	2407	6	294	0	0.1790	Image
birds	645	19	258	2	0.053	Audio
enron	1702	53	0	1001	0.064	Text

**Table 2:** Performance comparison of nine algorithms on the emotions dataset.

Algorithm	Hamming Loss	Ranking Loss	One Error	Coverage	Average Precision
HOMER	0.2525(5)	0.3040(6)	0.4032(5)	2.5863(6)	0.6897(6)
LP	0.2808(6)	0.3393(7)	0.4605(7)	2.6669(7)	0.6643(7)
RAkEL	0.2153(3)	0.1891(4)	0.3120(3)	1.9464(4)	0.7758(4)
Rank-SVM	0.3713(9)	0.4273(9)	0.6154(9)	3.0264(8)	0.5714(9)
BP_MLL	0.3519(8)	0.4143(8)	0.5868(8)	3.0759(9)	0.5732(8)
CC	0.2171(4)	0.1729(3)	0.3124(4)	1.8134(3)	0.7852(3)
CCE	0.2064(2)	0.1646(2)	0.2719(2)	1.7699(2)	0.8001(2)
LLSF-DL	0.2893(7)	0.2867(5)	0.4273(6)	2.3407(5)	0.6936(5)
PCC-FS	**0.2008(1)**	**0.1503(1)**	**0.2550(1)**	**1.7174(1)**	**0.8130(1)**

**Table 3:** Performance comparisons of nine algorithms on the CAL500 dataset.

Algorithm	Hamming Loss	Ranking Loss	One Error	Coverage	Average Precision
HOMER	0.2104(8)	0.4071(8)	0.8565(8)	169.3291(8)	0.2609(8)
LP	0.1993(7)	0.6559(9)	0.9880(9)	171.1590(9)	0.1164(9)
RAkEL	0.1686(6)	0.2870(7)	0.3529(7)	165.3036(7)	0.4008(7)
Rank-SVM	0.1376(2)	0.1824(5)	0.1156(2)	129.2589(2)	0.4986(4)
BP_MLL	0.1480(5)	0.1815(4)	0.1186(3)	130.084(6)	0.4967(5)
CC	0.1386(4)	0.1814(3)	0.1216(4)	129.698(4)	0.5025(3)
CCE	0.1377(3)	0.1781(2)	0.1255(5)	129.851(5)	0.5094(2)
LLSF-DL	0.2330(9)	0.1985(6)	0.3500(6)	**126.967(1)**	0.4463(6)
PCC-FS	**0.1370(1)**	**0.1766(1)**	**0.1155(1)**	129.616(3)	**0.5125(1)**

**Table 4:** Performance comparisons of nine algorithms on the yeast dataset.

Algorithm	Hamming Loss	Ranking Loss	One Error	Coverage	Average Precision
HOMER	0.2619(8)	0.3287(8)	0.2871(7)	9.2457(8)	0.6259(8)
LP	0.2768(9)	0.3977(9)	0.5143(9)	9.3607(9)	0.5733(9)
RAkEL	0.2270(6)	0.2143(6)	0.2946(8)	7.5086(6)	0.7144(6)
Rank-SVM	0.2450(7)	0.1928(5)	0.2521(5)	6.3554(3)	0.7217(5)
BP_MLL	0.2112(5)	0.1761(4)	0.2429(4)	6.5101(5)	0.7473(4)
CC	**0.1993(1)**	0.1699(3)	0.2238(2)	6.4200(4)	0.7596(3)
CCE	0.2015(3)	0.1679(2)	0.2284(3)	6.3475(2)	0.7645(2)
LLSF-DL	0.2019(4)	0.2585(7)	0.2790(6)	8.5881(7)	0.7004(7)
PCC-FS	0.2013(2)	**0.1665(1)**	**0.2156(1)**	**6.3335(1)**	**0.7666(1)**

**Table 5:** Performance comparisons of nine algorithms on the flags dataset.

Algorithm	Hamming Loss	Ranking Loss	One Error	Coverage	Average Precision
HOMER	0.2683(3)	0.2895(7)	0.3703(7)	4.1221(7)	0.7630(7)
LP	0.2962(6)	0.5011(8)	0.5587(8)	4.9495(8)	0.6407(8)
RAkEL	**0.2428(1)**	0.2332(5)	0.2255(3)	3.7824(3)	0.8118(2)
Rank-SVM	0.5931(9)	0.7052(9)	0.7618(9)	5.5303(9)	0.4987(9)
BP_MLL	0.3225(8)	0.2226(3)	0.2288(4)	3.8734(5)	0.8028(5)
CC	0.2785(5)	0.2136(2)	0.2344(5)	**3.7400(1)**	0.8117(3)
CCE	0.2749(4)	0.2252(4)	0.2283(2)	3.8513(4)	0.8032(4)
LLSF-DL	0.2987(7)	0.2786(6)	0.2838(6)	4.0921(6)	0.7683(6)
PCC-FS	0.2619(2)	**0.2068(1)**	**0.2135(1)**	3.7492(2)	**0.8214(1)**

**Table 6:** Performance comparisons of nine algorithms on the scene dataset.

Algorithm	Hamming Loss	Ranking Loss	One Error	Coverage	Average Precision
HOMER	0.1488(7)	0.2345(9)	0.4595(9)	1.2685(9)	0.6946(9)
LP	0.1439(6)	0.2121(8)	0.3984(7)	1.1550(8)	0.7308(8)
RAkEL	0.1014(4)	0.0998(4)	0.2672(4)	0.5854(4)	0.8378(4)
Rank-SVM	0.1501(8)	0.1039(5)	0.2863(5)	0.6266(5)	0.8275(5)
BP_MLL	0.1859(9)	0.1383(6)	0.4550(8)	0.7725(6)	0.7411(7)
CC	0.1137(5)	**0.0801(1)**	0.2439(2)	**0.4844(1)**	**0.8556(1)**
CCE	0.0949(2)	0.0922(3)	0.2447(3)	0.5439(3)	0.8495(3)
LLSF-DL	0.0998(3)	0.1898(7)	0.3482(6)	1.0536(7)	0.7579(6)
PCC-FS	**0.0940(1)**	0.0872(2)	**0.2401(1)**	0.5193(2)	0.8540(2)

**Table 7:** Performance comparisons of nine algorithms on the birds dataset.

Algorithm	Hamming Loss	Ranking Loss	One Error	Coverage	Average Precision
HOMER	0.0641(4)	0.2076(3)	0.8231(6)	5.2117(6)	0.3806(5)
LP	0.0731(5)	0.2733(7)	0.9009(9)	6.0743(8)	0.2673(6)
RAkEL	**0.0502(1)**	**0.1523 (1)**	0.6957(4)	4.0188(4)	0.5271(3)
Rank-SVM	0.1080(9)	0.5359(9)	0.8273(7)	6.2380(9)	0.2421(7)
BP_MLL	0.0587(3)	0.4486(8)	0.8637(8)	5.2591(7)	0.2023(9)
CC	0.0923(8)	0.2487(6)	0.4959(2)	3.3094(2)	0.5311(2)
CCE	0.0878(7)	0.2521(5)	0.5162(3)	3.3771(3)	0.5138(4)
LLSF-DL	0.0536(2)	0.1809(2)	0.8229(5)	4.2128(5)	0.2239(8)
PCC-FS	0.0827(6)	0.2265(4)	**0.4457(1)**	**3.1203(1)**	**0.5646(1)**

**Table 8:** Performance comparisons of nine algorithms on the enron dataset.

Algorithm	Hamming Loss	Ranking Loss	One Error	Coverage	Average Precision
HOMER	0.0606(7)	0.2471(7)	0.4918(7)	28.0953(7)	0.5067(7)
LP	0.0707(8)	0.5480(8)	0.8144(8)	39.5441(8)	0.2246(8)
RAkEL	0.0484(5)	0.2011(6)	0.2774(2)	25.2163(6)	0.6156(6)
Rank-SVM	0.0737(9)	0.6256(9)	0.8854(9)	45.4454(9)	0.1471(9)
BP_MLL	0.0553(6)	**0.0716(1)**	**0.2679(1)**	**11.2064(1)**	**0.6847(1)**
CC	0.0481(4)	0.0781(2)	0.3099(4)	11.8147(2)	0.6817(3)
CCE	0.0480(3)	0.0809(4)	0.3264(6)	12.0097(4)	0.6720(4)
LLSF-DL	**0.0454(1)**	0.1545(5)	0.2954(3)	20.9599(5)	0.6408(5)
PCC-FS	0.0474(2)	0.0784(3)	0.3152(5)	11.8254(3)	0.6832(2)

**Table 9:** Summary of the Friedman Statistics $F_{F}$ (k=9,N=7) and the critical value in terms of each evaluation criterion (k: #comparing algorithms; N: #datasets).

Evaluation Criteria	$F_{F}$	Critical Value (α=0.05)
Hamming Loss	4.2558	2.1382
Ranking Loss	8.9239	
One-Error	7.8679	
Coverage	9.6106	
Average Precision	13.6875	

**Table 10:** Comparisons between PCC-FS and other algorithms.

	Hamming Loss	Ranking Loss	One Error	Coverage	Average Precision
HOMER		√	√	√	√
LP	√	√	√	√	√
RAkEL					
Rank-SVM	√	√	√	√	√
BP_MLL	√				
CC					
CCE					
LLSF-DL					√
